# Lack of VEGFA/KDR Signaling in Conventional Renal Cell Carcinoma Explains the Low Efficacy of Target Therapy and Frequent Adverse Events

**DOI:** 10.3390/ijms25137359

**Published:** 2024-07-04

**Authors:** Lehel Peterfi, Maria V. Yusenko, Gyula Kovacs, Tamas Beothe

**Affiliations:** 1 Department of Urology, Medical School, University of Pecs, 7602 Pecs, Hungary; peltaao.pte@pte.hu; 2 Institute of Human Genetics, Ruhr-University, 44801 Bochum, Germany; maria.yusenko@rub.de; 3 Medical Faculty, Ruprecht-Karls-University, 69117 Heidelberg, Germany; 4 Department of Urology, Peterfy Sandor Hospital, 1076 Budapest, Hungary; beothe.tamas@peterfykh.hu

**Keywords:** VEGFA, KDR, immunohistochemistry, renal cell carcinoma, adverse events

## Abstract

It is acknowledged that conventional renal cell carcinoma (cRCC), which makes up 85% of renal malignancies, is a highly vascular tumor. Humanized monoclonal antibodies were developed to inhibit tumor neo-angiogenesis, which is driven by VEGFA/KDR signaling. The results largely met our expectations, and in several cases, adverse events occurred. Our study aimed to analyze the expression of VEGFA and its receptor KDR by immunohistochemistry in tissue multi-array containing 811 cRCC and find a correlation between VEGFA/KDR signaling and new vessel formation. None of the 811 cRCC displayed VEGFA-positive immunostaining. However, each glomerulus in normal kidney showed VEGFA-positive endothelial cells. KDR expression in endothelial meshwork was found in only 9% of cRCC, whereas 2% of the cRCC displayed positive KDR reaction in the cytoplasm of tumor cells. Our results disclose the involvement of VEGFA/KDR signaling in the neo-vascularization of cRCC and explain the frequent resistance to drugs targeting the VEGFA/KDR signaling and the high frequency of adverse events.

## 1. Introduction

Conventional renal cell carcinoma (cRCC) arises from proximal tubular cells (PTCs) of the kidney, which differentiate from embryonic mesenchymal blastema through mesenchymal-to-epithelium transition (MET) to become polarized epithelial cells [1]. Not only does the renal tubular system originate from blastemal cells, but also stromal endothelial cells, smooth muscle cells, and fibroblasts. On the other hand, carcinomas of other organs develop from ento- or ectodermal epithelial cells and their vascular support comes from their parental stroma of mesodermal origin. This fact may explain the differences in the biology of renal “carcinomas” in general and in the carcinomas of other organs.

The aggressive growth of cancer cells relies not only on their biological behavior, but also on their communication with the inflammatory tumor microenvironment (TME) [2,3]. The TME comprises newly formed blood vessels, permanently activated cancer-associated fibroblasts (CAFs) secreting growth factors and cytokines, and innate and adaptive immune cells, all of which are embedded in the extracellular matrix (ECM) [4]. The development of new blood vessels is essential for tumor growth, especially at the metastatic site [5]. The vascular endothelial growth factor (VEGF) and kinase insert domain receptor (KDR) signaling has been identified as a key mediator of neo-vascularization and target of therapy [6].

As cRCC is a highly vascular tumor, over the past years, the treatment of RCC has been focused on the inhibition of VEGFA/KDR signaling pathway with an increasing number of new drugs, including tyrosine kinase inhibitors as well as anti-VEGFA humanized monoclonal antibody [7]. However, durable responses are rare and most patients develop resistance to anti-angiogenic agents after months of treatment [8]. Moreover, prolonged treatment is associated with significant toxicities and adverse events (AEs) [9,10,11].

Although blocking the VEGFA/KDR signaling with antibody therapy has been used for the treatment of cRCC for more than ten years, no immunohistochemical analysis of the expression and cellular localization of VEGFA and KDR proteins has been carried out. Previously, our global gene expression analysis by Affymetrix Human Genome U133 Plus 2.0 array displayed the expression of VEGF and its receptor KDR exclusively in cRCC. As RNA expression does not necessarily correspond to protein expression, we have analyzed the occurrence and cellular location of VEGFA and KDR proteins by immunohistochemistry in 811 cRCC. The objective of this study was to evaluate the expression of VEGFA and KDR proteins in cRCC and their correlation with neo-angiogenesis and postoperative tumor relapse.

## 2. Results

### 2.1. RNA Expression Profile

**Figure 1:**
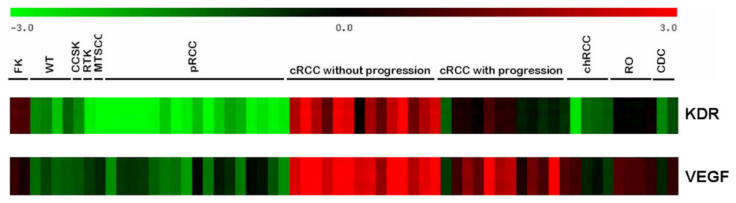
Expression of KDR and VEGF mRNA in distinct types of kidney tumors. KDR mRNA is expressed exclusively in cRCC without progression, whereas VEGF is expressed in nearly all conventional RCCs (marked in red). Abbreviations: FK, fetal kidney; WT, Wilms’ tumor; CCSK, clear cell sarcoma of the kidney; RTK, rhabdoid tumor of the kidney; MTSCC, mucinous tubular and spindle cell carcinoma; pRCC, papillary renal cell carcinoma; cRCC, conventional renal cell carcinoma; chRCC, chromophobe RCC; RO, renal oncocytoma; CDC, collecting duct carcinoma.

Gene expression profiles of 12 cRCCs without progression during the eight-year follow-up and 12 cRCCs with progression within 3 years after tumor nephrectomy were established according to the Affymetrix protocol. The diagnosis of cRCC was confirmed by genetic analysis, where each tumor displayed a chromosome 3p deletion. By applying the gene set enrichment analysis (GSEA), the overexpression of several genes was detected in cRCC. KDR mRNA was expressed exclusively in cRCC without progression, whereas VEGF expression was seen in nearly all the cRCCs included in this study (Figure 1). No increased expression of VEGF or KDR was seen in other types of kidney tumors. As mRNA expression does not necessary corresponds to protein expression, next immunohistochemistry was applied to detect the occurrence and cellular localization of VEGFA and KDR in tumor tissues.

### 2.2. Expression of VEGFA and KDR in Normal Kidney

**Figure 2:**
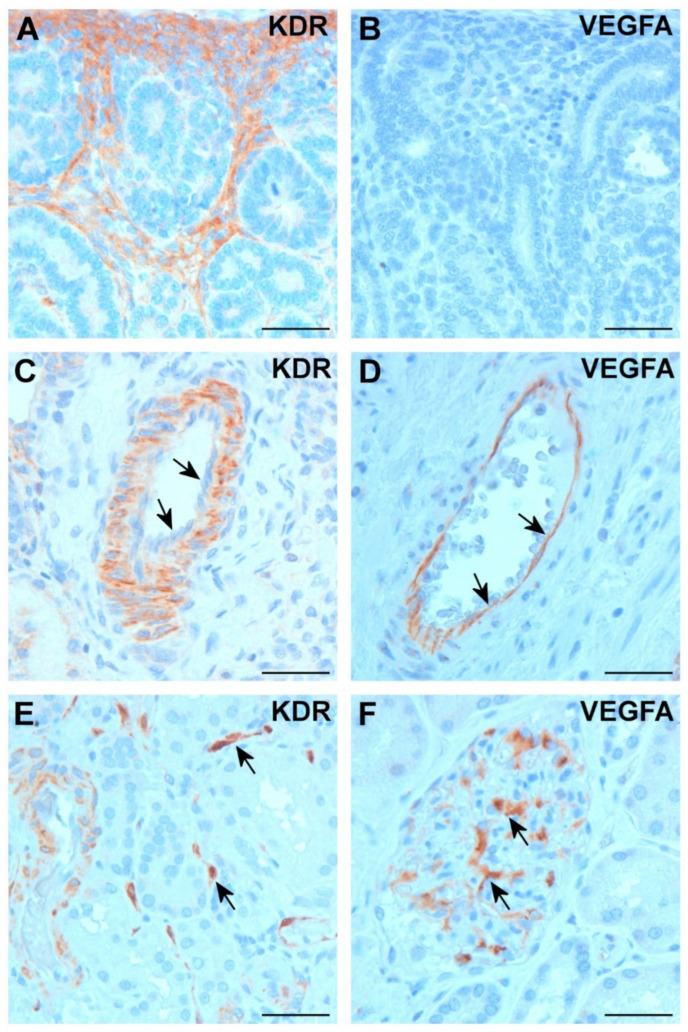
Expression of KDR and VEGFA in fetal and adult kidneys. (**A**) KDR is expressed in emerging stromal fibroblasts in fetal kidney. (**B**) No VEGFA expression was detected in fetal kidney. (**C**) In adult kidney, larger blood vessels displayed positive KDR staining in myo-fibroblasts of arterial walls, whereas endothelial cells were negative (arrows). (**D**) VEGFA expressed exclusively in endothelial cells of an arteria of similar caliber (arrows). (**E**) In some cortical areas of adult kidney, single endothelial cells were positive with KDR (arrows). (**F**) VEGFA expressed exclusively in endothelial cells of glomeruli (arrows). Scale bar: 40 μm.

In fetal kidneys of 10 to 17 weeks of gestation, strong KDR immunostaining was seen in fibroblast-like mesenchymal cells around the ureteric bud tip, which are progenitors of stromal fibroblasts, smooth muscle, and endothelial cells in fetal and adult kidneys (Figure 2A). Smooth muscle cells and myo-fibroblasts in the walls of small arteria in the medullary area were also positive for KDR antibody. No VEGFA staining was seen in fetal kidneys (Figure 2B).

In adult kidney, myo-fibroblasts in the wall of arteries showed strong KDR positivity, whereas the endothelial cells of these vessels remained unstained (Figure 2C). In the kidney medulla, capillaries running parallel with the collecting ducts showed a weak positive staining with KDR. VEGFA immunohistochemistry displayed a positive staining of endothelial cells of arteries (Figure 2D). Occasionally, star-like endothelial cells in normal kidney stroma showed a weak positive staining with KDR (Figure 2E). The VEGFA protein was expressed in each glomerulus of adult kidneys (Figure 2F). No KDR or VEGFA expression was seen in stromal fibroblasts or renal tubular cells.

### 2.3. Expression of VEGFA and KDR in Tumor Tissues

**Figure 3:**
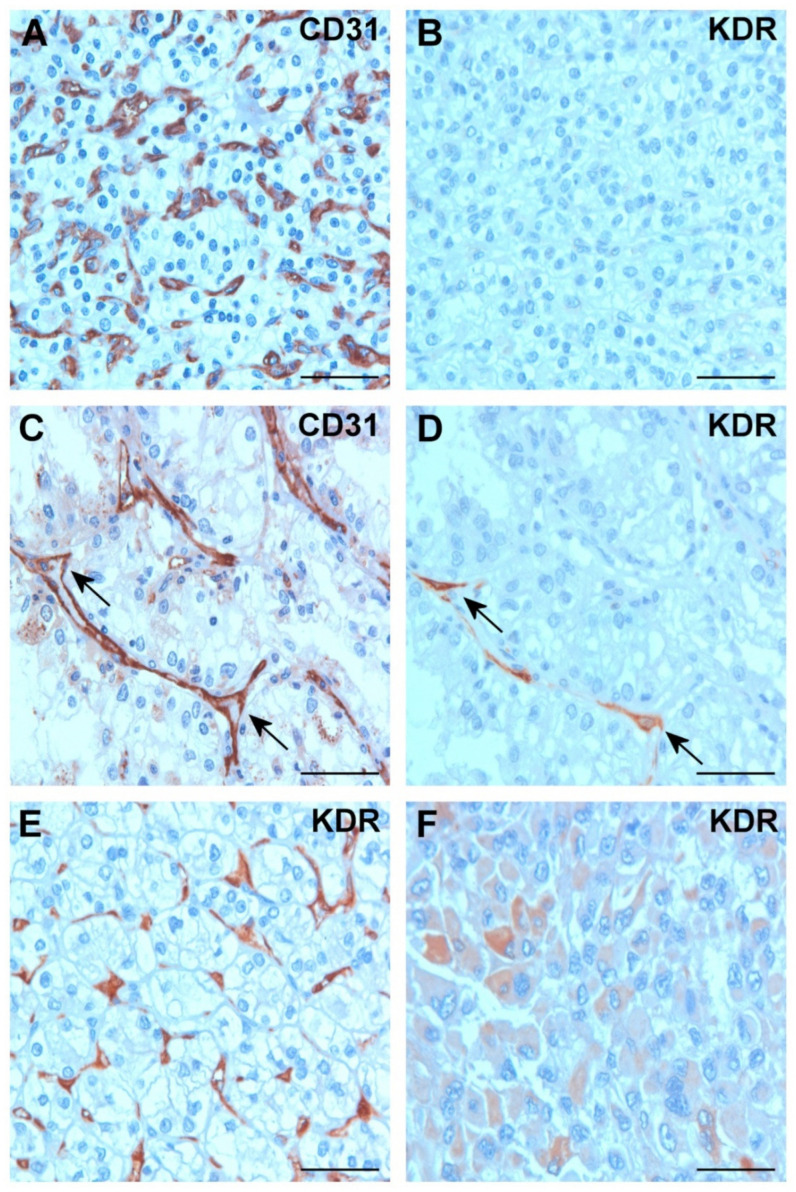
Expression of CD31 and KDR in conventional RCC. (**A**) Conventional RCC showing a capillary meshwork of CD31-positive endothelial cells. (**B**) No KDR expression is seen in identical TMA-biopsy specimen. (**C**) Strong CD31 expression in endothelial cells of a tubular–papillary growing conventional RCC (arrows). (**D**) In the same tumor biopsy, only few endothelial cells display positive staining with KDR antibody (arrows). (**E**) Diffuse regular meshwork of KDR-positive capillaries in a conventional RCC. (**F**) Positive KDR staining in the cytoplasm of a cRCC of rhabdoid histology. Scale bar: 40 μm.

None of the 811 cRCCs displayed a positive VEGFA immunoreaction either in stromal endothelial or tumor cells. However, each glomerulus of normal adult kidney and those placed in TMA displayed VEGFA-positive staining of endothelial cells, confirming the specificity of VEGFA antibody. KDR expression was seen only in 90 of 811 cRCCs. To improve the vascular pattern of KDR-positive and -negative tumors, an identical set of TMAs was stained with CD31 antibody. All KDR-negative tumors displayed CD31 positivity of the fine endothelial network of cRCC (Figure 3A,B). Larger and partly dilated vessels were seen in 51 cRCCs where all endothelial cells stained positive with the CD31 antibody (Figure 3C). However, only few endothelial cells were positive with the KDR antibody (Figure 3D). Only 22 cRCCs showed positive staining of the fine endothelial meshwork with the KDR antibody (Figure 3E). And finally, in 17 cRCCs, scattered cytoplasmic KDR-positive staining was seen (Figure 3F).

### 2.4. Expression of KDR in Tumor Cells

Multivariate Cox proportional hazard regression analysis failed to show an independent prognostic significance of KDR expression. Kaplan–Meier analysis revealed that the estimated survival of patients with cRCC showing focal or diffuse KDR positivity and those without KDR expression does not differ. Only patients with KDR positive tumor cells have a shorter disease-free survival compared to those without or with focal and diffuse vascular KDR expression (*p* = 0.015). Considering that only 2% of the 811 cRCCs showed tumor cell positivity, this finding has no clinical relevance.

## 3. Discussion

It was suggested four decades ago that neo-angiogenesis is a requirement for the growth of malignant tumors [5]. Tumor xenograft models demonstrated that the development of new blood vessels from the host tissues is essential for growth and progression of cancer cells and confirmed the importance of neo-angiogenesis at least in the case of transplanted tumors [12,13,14]. One of the main regulators of angiogenesis is the VEGFA/KDR signaling axis [6]. Therefore, pharmaceutical research was focused on developing new drugs to target tumor neo-angiogenesis with the intent of blocking the growth of new vessels. Several VEGFA/KDR inhibitors have been approved by the US Food and Drug Administration (FDA) in recent years.

The humanized monoclonal antibody bevacizumab blocks the binding of VEGFA to its receptor KDR, inhibiting the VEGFA/KDR signaling and new vessel formation [11]. The bevacizumab (Avastin) was approved in 2008 for the treatment of metastatic breast cancer, but was revoked three years later because it did not show an effect on progression-free survival and instead showed serious adverse effects (AEs), including death [15,16]. Despite the lack of therapeutic advantage and serious AEs, bevacizumab has been used to treat other types of cancers. A study of bevacizumab therapy in 1039 patients with advanced pancreas, breast, and prostate cancer resulted in grade 2 or higher AEs, such as hypertension (15.0–50.5%), proteinuria (6.4–30.5%) and composite toxicity (19.4–61.9%) [17]. Bevacizumab therapy resulted in grades 3–5 hemorrhage, leading to a mortality rate of 6% in patients with non-small-cell lung cancer [18]. Tyrosine kinase inhibitor monoclonal antibodies such as sunitinib and sorafenib block the downstream signaling through KDR. Several randomized, placebo-controlled phase III trials on distinct types of cancers with the new drugs showed low efficacy and occasionally AEs [19].

It was acknowledged at the beginning of the anti-angiogenic therapy of RCC with bevacizumab that long-term responses are rare, and that several tumors developed resistance and progressed [8,20]. Despite these disappointing results, intensive studies were assigned to test the efficacy of new anti-VEGFA treatment of cRCC [21,22]. More positive results without AEs were achieved in the treatment of metastatic cRCC by the tyrosine kinase inhibitors sunitinib and sorafenib, as was shown in several phase II–IV trials [23]. This can be explained by the fact that multi-kinase inhibitors target not only the KDR but also PDGFR, c-kit, and RET, and that sunitinib and sorafenib treatment does not affect the nitrogen oxide (NO) synthesis in normal endothelial cells and the interactions between podocytes and glomerular endothelial cells. Therefore, much fewer and milder AEs have been observed, for example, in most cases fatigue and hand-foot syndrome.

Of interest, no comprehensive analysis has been carried out to confirm the expression and cellular localization of VEGFA and KDR proteins in cRCC. A previous study applying immunohistochemistry detected positive staining for KDR in 243 of 262 vascular tumors, but none of the 51 renal cell carcinomas displayed KDR expression [24]. The focal or diffuse expression of KDR in endothelial cells in 9% of cRCC as shown in our study is insufficient for a successful tyrosine kinase inhibitor therapy. Moreover, the vast majority of the 73 cRCCs showing KDR positivity in endothelial cells were slowly growing tumors with a fine vascular network showing grade 1 (73%), pT1 (80%), and stage I (78%), which rarely metastasize.

Our observation may explain not only the disappointing results of anti-VEGFA therapy of cRCC, but also the frequent AEs posing a threat to patient health and quality of life. In a double-blinded AVOREN trial, hypertension (7%) and proteinuria (4%) were the most common grade 3 or 4 AE associated with bevacizumab therapy [25]. Further, the three or four frequent AEs are bleeding (3%), thromboembolic events (3%), gastrointestinal perforation (1%), and impaired wound healing (1%) [26]. Hypertension is one of the best documented and most frequently observed AEs of systemic inhibition of VEGF signaling pathway [27]. VEGFA inhibition diminishes NO synthesis in normal endothelial cells, resulting in vasoconstriction and increased blood pressure [28]. We showed that VEGFA is expressed in glomeruli in normal adult kidneys but not in endothelial cells of the vascular meshwork of cRCC. Therefore, anti-VEGFA therapy cannot affect neo-angiogenesis or the growth of cRCC but rather through the inhibition of VEGF-dependent interactions between podocytes and glomerular endothelial cells, which disrupt the filtration barrier and lead to proteinuria [29,30].

The results presented here show the lack of VEGFA expression in tumor endothelial cells and disclose the role of VEGFA/KDR signaling in neo-vascularization of cRCC. This finding explains not only the frequent resistance to drugs targeting the VEGFA/KDR signaling but also the high frequency of AEs such as hypertension and proteinuria. In any anti-cancer therapy, one should first ask the following question: what do we target? It is unacceptable to apply any target therapy without showing the presence of the target as it was conducted with bevacizumab for cRCC. We suggest that before starting to target a gene in question, its expression and exact cellular localization in the tumor tissue should be confirmed by immunohistochemistry.

## 4. Materials and Methods

### 4.1. Microarray-Based Gene Expression Analysis

Gene expression analysis of distinct types of RCCs were carried out as described earlier [31]. The correct diagnosis of each tumor has been confirmed by genetic analysis. The expression profile data have been deposited in NCBI Gene Expression Omnibus under accession number GSE11151.

### 4.2. Patients and Tissue Samples

Tissue samples of cRCCs obtained from patients operated consecutively between 2000 and 2015 in the Department of Urology, Medical School, University of Pecs, Hungary. In the cohort of 811 patients included in this study, the male/female ratio was 481 (59%) to 330 (41%), the mean age was 60.7 ± 11.3 years with a range of 20–88 years, and the average size of tumors was 52.8 ± 29.1 mm. At the time of surgery, metastatic tumor growth was detected in 73 (9%) patients, whereas postoperative tumor relapse occurred in 119 patients (15%) during the median follow-up of 62 ± 30 months.

Tumors were diagnosed according to the Heidelberg Classification and 3-tiered grading system [32,33]. The latest TNM classification was applied [34]. Three to five core biopsies with a diameter of 0.6 mm were taken from each tumor and placed in recipient block using a Manual Tissue Arrayer (MTA1, Beecher Instruments, Inc., Sun Prairie, WI, USA). Fetal and adult kidneys were included in the study and placed in each TMA. Consecutive TMA slides were used for IHC to be able to compare the staining in the same tumor areas.

### 4.3. Immunohistochemistry

The epitope retrieval of 4 μm thick dewaxed sections was carried out in citrate buffer (pH 6) in 2100-Retriever (Pick-Cell Laboratories, Amsterdam, The Netherlands). For blocking the endogenous peroxidase, the slides were covered with Envision FLEX Peroxidase Blocking Reagent for 10 min and were incubated at room temperature for one hour with the following antibodies. Rabbit monoclonal anti-VEGFA antibody (EP1176Y, ab52917, abcam, Cambridge, UK), which was developed against the aa200 to the C-terminus of VEGFA, and which was applied at a dilution of 1:100. Monoclonal anti-KDR antibody (B.309.4, In Vitrogen, Budapest, Hungary), which was developed against the 150 amino acid carboxy-terminal residues of human VEGF receptor 2, and which was applied at a dilution of 1:400. The specificity of both antibodies was verified with Western blotting. Envision FLEX horseradish–peroxidase conjugated secondary antibody (DAKO) was applied for 30 min at room temperature and color was developed using the DAB (3,3′-diaminobenzidin) (DAKO). Tissue sections were counterstained with Mayer’s hematoxylin (Lillie’s modification, DAKO), and after 10 s bluing in ammonium hydroxide solution, they were mounted with Glycergel (DAKO). For negative control, the primary antibody was omitted.

To identify blood vessels in the biopsies, one set of slides was analyzed for the expression of CD31. Slides were incubated with mouse anti-CD31 monoclonal antibody (JC70A, DAKO/Agilent, Budapest, Hungary) at room temperature for 70 min. Detection was performed using the Novolink polymer kit (Leica Biosystems/Novocastra, Budapest, Hungary). The immunohistochemistry was executed by a four-channel TECAN Freedom Evo liquid handling platform (Mannedorf, Switzerland).

Photographs were taken by a Leitz DMRBE microscope, equipped with HC PLAN APO 20 × 0.70 microscope objective, and a ProgRes C14 camera.

### 4.4. Statistical Analysis

Correlations between categorical variables were estimated with Fisher’s exact test. The Kaplan–Meier method was used for estimation of the cumulative survival distributions. For establishing the differences between the groups, we applied the log-rank test. To detect the significance of clinical–pathological variables and KDR expression, the univariate and multivariate COX hazard regression model was applied. The analysis was performed with the IBM SPSS Statistics v.25 for Windows (Inc., Chicago, IL, USA). The *p*-value < 0.05 was considered as the limit of statistical significance.

## Data Availability

The material and datasets used and/or analyzed during the current study are available from Gyula Kovacs, Department of Urology, Munkacsy M utca 2, 7621 Pecs, Hungary. E-mail: g.kovacs@gmx.de.
